# Optogenetic stimulation of the brainstem dorsal motor nucleus ameliorates acute pancreatitis

**DOI:** 10.3389/fimmu.2023.1166212

**Published:** 2023-04-25

**Authors:** Dane A. Thompson, Tea Tsaava, Arvind Rishi, Sandeep Nadella, Lopa Mishra, David A. Tuveson, Valentin A. Pavlov, Michael Brines, Kevin J. Tracey, Sangeeta S. Chavan

**Affiliations:** ^1^ Laboratory of Biomedical Sciences, Institute for Bioelectronic Medicine, Feinstein Institutes for Medical Research, Northwell Health, Manhasset, NY, United States; ^2^ The Elmezzi Graduate School of Molecular Medicine, Manhasset, NY, United States; ^3^ Department of Surgery, Northshore University Hospital, Northwell Health, Feinstein Institutes for Medical Research, Northwell Health, Manhasset, NY, United States; ^4^ Donald and Barbara Zucker School of Medicine at Hofstra/Northwell, Hofstra University, Hempstead, NY, United States; ^5^ Department of Pathology and Laboratory Medicine, Donald and Barbara Zucker School of Medicine at Hofstra/Northwell, Hempstead, NY, United States; ^6^ Cold Spring Harbor Laboratory, Cold Spring Harbor, NY, United States; ^7^ Lustgarten Foundation Pancreatic Cancer Research Laboratory, Cold Spring Harbor, NY, United States

**Keywords:** acute pancreatitis, dorsal motor nucleus, vagus nerve, inflammation, cytokines, cholinergic anti-inflammatory pathway (CAP)

## Abstract

**Introduction:**

Inflammation is an inherently self-amplifying process, resulting in progressive tissue damage when unresolved. A brake on this positive feedback system is provided by the nervous system which has evolved to detect inflammatory signals and respond by activating anti-inflammatory processes, including the cholinergic anti-inflammatory pathway mediated by the vagus nerve. Acute pancreatitis, a common and serious condition without effective therapy, develops when acinar cell injury activates intrapancreatic inflammation. Prior study has shown that electrical stimulation of the carotid sheath, which contains the vagus nerve, boosts the endogenous anti-inflammatory response and ameliorates acute pancreatitis, but it remains unknown whether these anti-inflammatory signals originate in the brain.

**Methods:**

Here, we used optogenetics to selectively activate efferent vagus nerve fibers originating in the brainstem dorsal motor nucleus of the vagus (DMN) and evaluated the effects on caerulein-induced pancreatitis.

**Results:**

Stimulation of the cholinergic neurons in the DMN significantly attenuates the severity of pancreatitis as indicated by reduced serum amylase, pancreatic cytokines, tissue damage, and edema. Either vagotomy or silencing cholinergic nicotinic receptor signaling by pre-administration of the antagonist mecamylamine abolishes the beneficial effects.

**Discussion:**

These results provide the first evidence that efferent vagus cholinergic neurons residing in the brainstem DMN can inhibit pancreatic inflammation and implicate the cholinergic anti-inflammatory pathway as a potential therapeutic target for acute pancreatitis.

## Introduction

Acute pancreatitis is a common inflammatory condition leading to over 250,000 hospitalizations each year in the United States alone (1). In addition to the significant morbidity associated with acute pancreatitis, up to 20% of patients develop pancreatic necrosis, which results in a mortality rate of 15%-30% (2). Most often the result of alcohol or gallstones, pancreatitis pathogenesis begins with acinar cell damage and inappropriate activation of digestive enzymes, which subsequently initiate inflammatory responses (3–5). Release of damage-associated molecular patterns, e.g., high mobility group box 1 (HMGB1), stimulates acinar cells and resident pancreatic macrophages to release pro-inflammatory cytokines, chemokines (6–8), and other inflammatory mediators. Although these signals initially amplify inflammation, they are also essential for recruiting immune cells to clear tissue debris and for orchestrating healing.

However, the initial response to tissue injury can also fuel an inflammatory positive feedback loop, producing progressive local and systemic inflammation, further damaging tissue, and resulting in significant morbidity and mortality (9, 10). In order to mitigate maladaptive inflammatory responses, evolution has equipped mammals with a nervous system capable of sensing both local and systemic inflammation and of responding *via* multiple distinct neural pathways. Signals transmitted through the vagus nerve have been implicated in the regulation of inflammation (11–13). This pathway, termed the inflammatory reflex, is initiated by inflammatory mediators which stimulate sensory, afferent neurons in the vagus nerve. The resulting signals are transmitted to the brainstem nucleus tractus solitarius (NTS). Efferent signals originating in the dorsal motor nucleus of the vagus (DMN) are subsequently transmitted by vagus nerve cholinergic fibers, which stimulate splenic nerve activity (14), releasing norepinephrine within the spleen. Subsequently, a subset of T-cells is activated, which in turn signals to splenic macrophages *via* an alpha-7 nicotinic acetylcholine receptor (α7 nAChR)-dependent mechanism, resulting in reduced proinflammatory cytokine production (15–17). This motor arm of the inflammatory reflex is termed the cholinergic anti-inflammatory pathway (18, 19). An alternative anti-inflammatory neural pathway is also activated by afferent vagus nerve signaling through the NTS but this results in the release of norepinephrine by splanchnic sympathetic nerves, which inhibits immune cell activation *via* the β2 adrenergic receptor (20). An additional neural pathway also exists which is independent of the vagus nerve. In this system, afferent signaling initiated by inflammatory signals carried within the circulation results in sympathetic splanchnic nerve-mediated anti-inflammatory responses (21).

The vagus nerve widely innervates the viscera, including the pancreas, which is supplied by both sensory and motor fibers. Vagus sensory afferent neurons, residing in the bilateral nodose ganglia, innervate both endocrine and exocrine pancreas structures (22). Vagus motor efferent neurons are the important physiological regulators of both the endocrine and exocrine pancreas. Stimulation of these cholinergic neurons activates insulin secretion by beta cells, the release of gastrointestinal hormones, such as pancreatic polypeptide (23) and calcitonin gene-related peptide (CGRP) (24), and digestive enzymes from acinar cells (25). However, despite the well-established functionality of the vagus nerve in normal pancreatic physiology, its role in acute pancreatitis is unknown.

Prior study has demonstrated that a disruption of the vagus nerve *via* cervical vagotomy increases the severity of acute pancreatitis (26). Conversely, electrical stimulation of the carotid sheath containing the vagus nerve, as well as glossopharyngeal and sympathetic nerve fibers also known to mediate anti-inflammatory activity, reduces the severity of pancreatitis (27). As vagotomy and electrical stimulation affect both afferent and efferent fibers simultaneously, these methods cannot reveal the independent contributions of afferent and efferent pathways to anti-inflammatory effects in the pancreas. Pharmacological studies have demonstrated that acetylcholine, the neurotransmitter of the cholinergic efferent vagus nerve, can play a protective role in acute pancreatitis. Administration of the non-selective nicotinic receptor antagonist mecamylamine increased the severity of acute pancreatitis, whereas administration of the α_7_nAChR agonist GTS-21 decreased the severity of acute pancreatitis (26).

Although these findings suggest that vagus nerve signaling provides protective effects in acute pancreatitis, given the presence of multiple neuroimmune anti-inflammatory pathways it is currently unclear whether signals originating in the brain and transmitted *via* vagus efferent neurons regulate the severity of pancreatitis. Using optogenetics and a well-established acute pancreatitis model, here we demonstrate that cholinergic neurons residing in the brainstem DMN control pancreatic inflammation in a vagus nerve- and nAChR -dependent mechanism.

## Results

### Selective activation of DMN cholinergic neurons induces bradycardia

**Figure 1:**
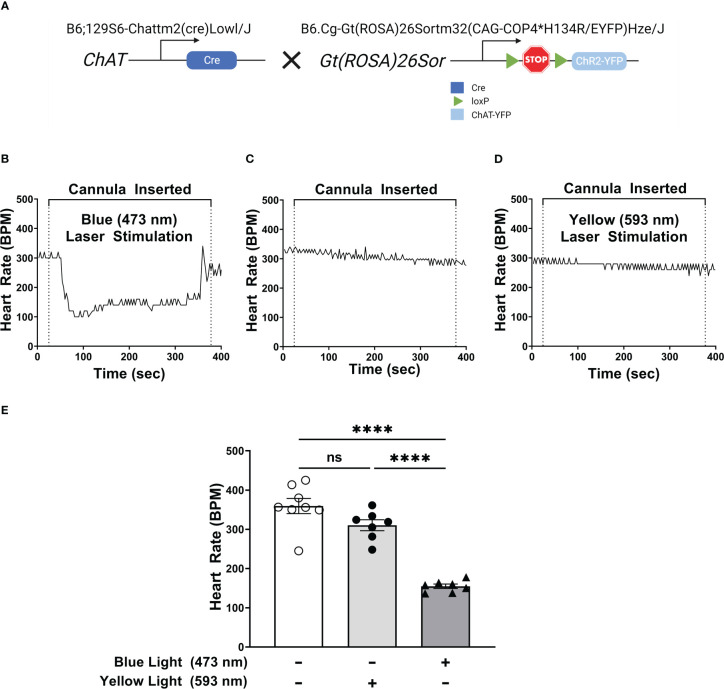
Selective activation of DMN cholinergic neurons invokes efferent vagus nerve activity, reducing heart rate. **(A)** Breeding strategy used to generate ChAT-ChR2-YFP mice which express a photosensitive channelrhodopisin in ChAT-positive cells, including cholinergic neurons. **(B–D)** Optogenetic stimulation of DMN cholinergic neurons produced a significant decrease in heart rate in ChAT-ChR2-eYP mice. A fiber optic cannula was placed in the left DMN of ChAT-ChR2-YFP mice and animals were then subjected to **(B)** blue light (473nm, 20Hz, 25% duty cycle, 8-12 mW, 5 minutes), **(C)** no light, or **(D)** yellow light (593.5nm, 20Hz, 25% duty cycle, 8-12 mW, 5 minutes) stimulation. A decrease in heart rate was observed during blue laser stimulation. Representative heart rate data is shown for each condition. **(E)** Stimulation of ChR2-expressing DMN cholinergic neurons with blue light, but not with yellow light, induces bradycardia in ChAT-ChR2-eYFP mice. Data are represented as individual mouse data points, which represent an average heart rate over the stimulation period, with mean ± SEM. One-way ANOVA with Tukey’s multiple comparison test, ****P ≤ 0.0001, ns, not significant.

Optogenetic stimulation of DMN cholinergic neurons has been shown to be protective during endotoxemia by reducing TNF production (28). However, the potential anti-inflammatory function of these neurons in controlling pancreatic inflammation arising from acute pancreatitis is unknown. To selectively activate the cholinergic neurons, we generated transgenic ChAT-Cre/ChR2-eYFP mice that express light-sensitive channelrhodopsin-2 (ChR2) (29) coupled to an enhanced yellow fluorescent protein (ChR2-eYFP) directed by the choline acetyltransferase (ChAT) promoter (**Figure 1A**). ChAT is an essential enzyme required for acetylcholine synthesis and is selectively expressed in cholinergic neurons in the central and peripheral nervous systems (30).

Because stimulation of efferent signaling in the vagus nerve has been associated with bradycardia (31, 32), we confirmed optogenetic stimulation of cholinergic neurons in the DMN by measuring heart rate during light stimulation. Selective stimulation using 473 nm light in the left DMN induced bradycardia for the duration of stimulation (**Figures 1B, E**). In contrast, the placement of a fiber optic cannula in the DMN for the duration of stimulation, without any light stimulation, failed to induce significant changes in heart rate (**Figures 1C, E**). Photostimulation of the DMN using yellow light (593.5nm), which does not activate ChR2, also failed to induce any significant change in heart rate in ChAT-Cre/ChR2-eYFP mice (**Figures 1D, E**). Photostimulation of DMN neurons with light at 473 or 593 nm also failed to induce bradycardia in littermate controls (non-carriers) not expressing ChR2-eYFP (**Supplemental Figures 1A–D**). Thus, exposure of the DMN to 473 nm light activates cholinergic neurons and induces bradycardia in ChAT-Cre/ChR2-eYFP mice.

### Selective activation of DMN cholinergic neurons attenuates disease severity during acute pancreatitis

**Figure 2:**
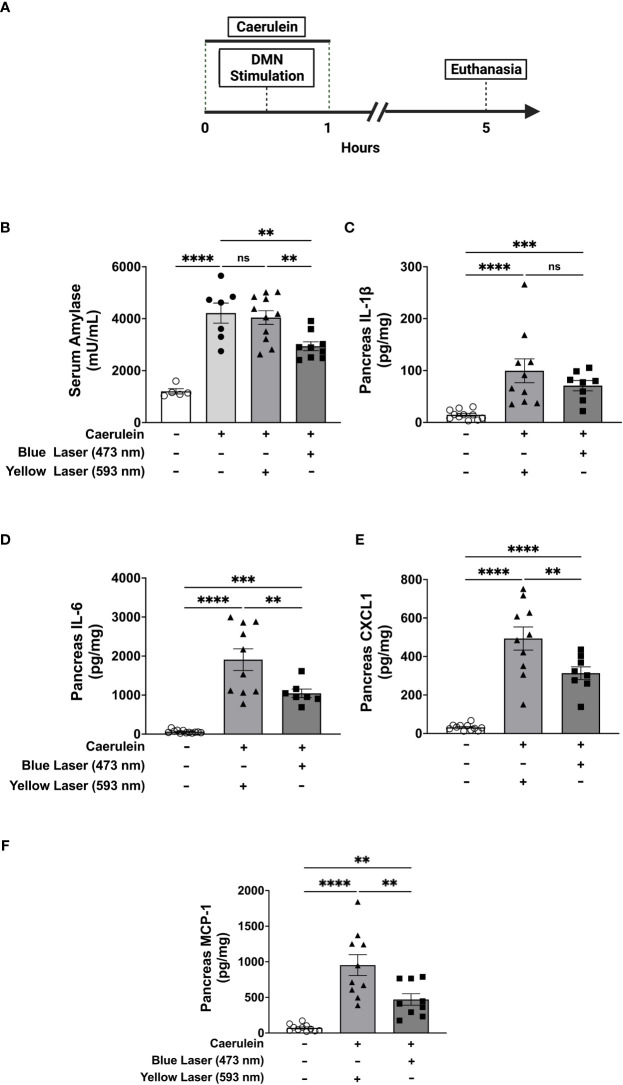
Selective activation of DMN cholinergic neurons reduces circulating and pancreatic markers of acute pancreatitis Acute pancreatitis was induced in ChAT-ChR2-YFP mice by two intraperitoneal injections of caerulein (50 µg/kg). A fiberoptic cannula was placed in the left DMN and mice were then subjected to blue light stimulation (473 nm, 20 Hz, 25% duty cycle, 8-12 mW, 5 minutes), yellow light stimulation (593.5 nm, 20 Hz, 25% duty cycle, 8-12 mW, 5 minutes), or no laser stimulation. Mice were euthanized 4 hours after the final dose of caerulein. **(A)** Compared to non-caerulein-injected controls, caerulein-injected mice show significantly increased serum amylase levels consistent with the induction of acute pancreatitis. Optogenetic stimulation with blue light, but not with yellow light, significantly decreases serum amylase levels. Data are represented as individual mouse data points with mean ± SEM. One-way ANOVA: no caerulein *vs* yellow laser *vs* no laser (****P ≤ 0.0001, n = 7-11). **(B)** Optogenetic stimulation with blue light decreases pancreatic levels of **(D)** IL-6, **(E)** CXCL-1, and **(F)** MCP-1, but not **(C)** IL-1β. Data are represented as individual mouse data points with mean ± SEM. One-way ANOVA, (**P ≤ 0.01, ***P ≤ 0.001, ****P ≤ 0.0001, n = 9-11). ns, not significant.

To determine the role of DMN cholinergic neurons in regulating acute pancreatitis, ChAT-ChR2 mice were subjected to two intraperitoneal injections of caerulein (50 µg/kg) one hour apart. Thirty mins following the first injection, animals underwent fiber optic cannula insertion in the DMN and were exposed to sham stimulation or light stimulation. Administration of caerulein induces pancreatitis as indicated by significant increases in serum amylase (**Figure 2A**). Selective stimulation of DMN cholinergic neurons in the DMN with 473 nm significantly attenuated serum amylase compared to the sham-stimulated group, whereas stimulation with yellow light at 593 nm, which does not activate ChR2, failed to attenuate serum amylase in ChAT-ChR2-eYFP mice (**Figure 2B**). Further, activation of DMN neurons using blue (473nm) or yellow (593nm) light failed to modulate serum amylase levels in littermate control ROSA-ChR2 mice that do not express ChR2 (**Supplemental Figure 2A**).

Acute pancreatitis is also characterized by increases in pancreatic pro-inflammatory cytokines and chemokines, including IL-1β, IL-6, IL-8, and monocyte chemoattractant protein-1 (MCP-1) which drive inflammation and tissue damage (33). In agreement with results obtained with serum amylase, the administration of caerulein induced significant increases in these pancreatic cytokines (**Figures 2C–F**). Selective activation of cholinergic neurons in the DMN with blue light (473 nm) significantly attenuated pancreatic pro-inflammatory cytokines as compared to the yellow light (593nm) stimulated controls. While pancreatic levels of pro-inflammatory cytokines were significantly elevated at the time point examined in this model of acute pancreatitis, circulating serum cytokine levels were much lower. Increases in IL-1β, IL-6, and CXCL-1 were observed after caerulein administrations as compared to naïve mice (**Supplemental Figures 3A–C**), but significant DMN cholinergic neurons-mediated suppression was only noted in CXCL1 (**Supplemental Figures 3A–C**). Thus, selective activation of brainstem DMN cholinergic neurons attenuates serum amylase and pancreatic pro-inflammatory cytokines levels in caerulein-induced acute pancreatitis.

**Figure 3:**
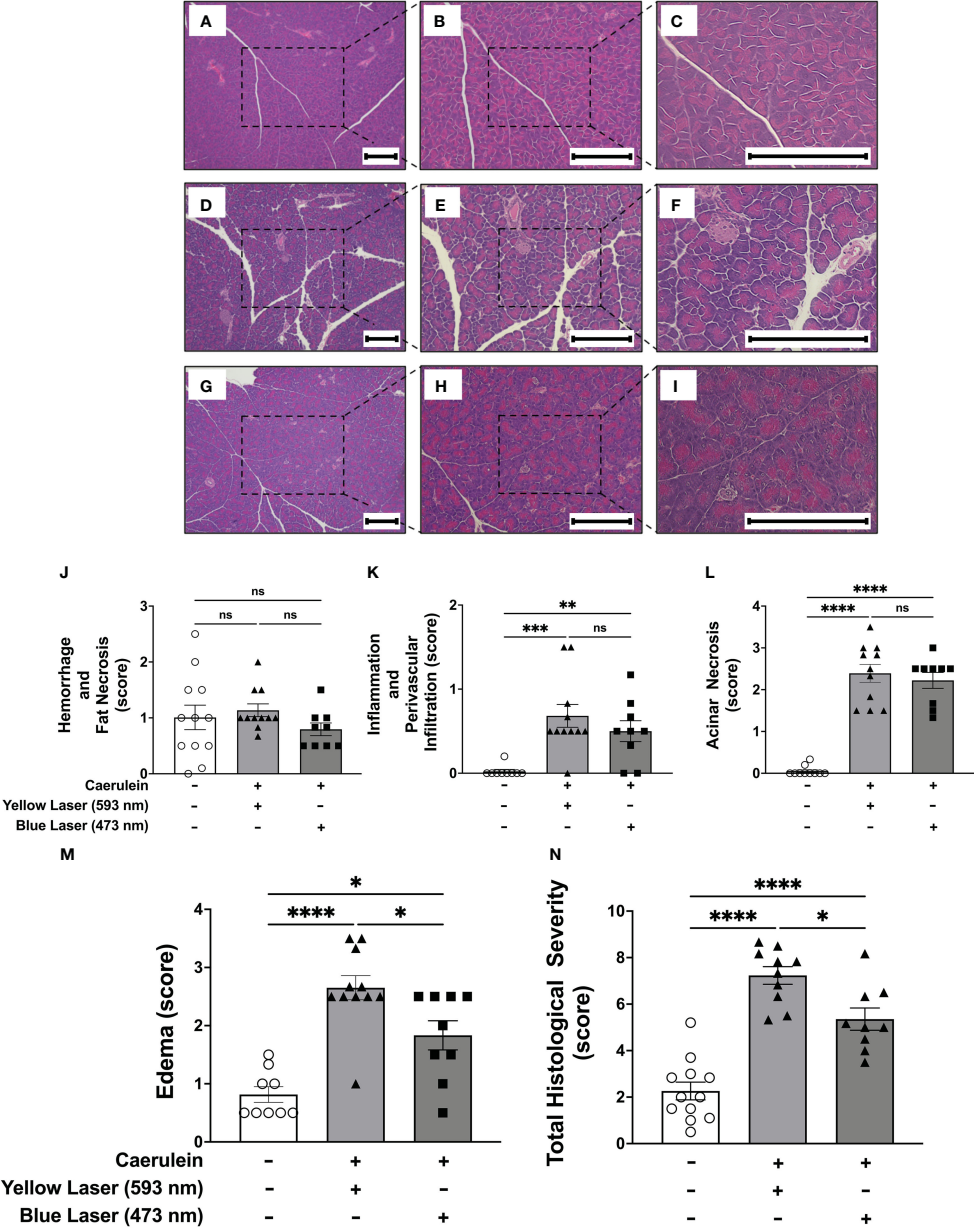
Selective activation of DMN cholinergic neurons reduces the histological severity of acute pancreatitis **(A–I)** Representative images of pancreatic tissue stained with H&E at 4x (left panels), 10x (middle panels), and 20x (right panels) from **(A–C)** non-caerulein-injected and non-stimulated controls, **(D–F)** caerulein-injected and yellow-light-stimulated, and **(G–I)**) caerulein-injected and blue light-stimulated mice. Bar = 200 μm. Histological scoring for **(J)** hemorrhage and fat necrosis, **(K)** acinar necrosis, **(L)** inflammation and perivascular infiltration, **(M)** edema, and **(N)** total severity. Data are represented as individual mouse data points with mean ± SEM. One way ANOVA with Kruskal-Wallis, *P P ≤ 0.05, **P ≤ 0.01, ***P ≤ 0.001, ****P ≤ 0.0001, n = 9-11, ns, not significant.

In addition to elevated pro-inflammatory cytokines, pancreatitis is also characterized by pancreatic tissue injury. Histopathologic examination of the pancreatic tissue demonstrated significant increases in acinar necrosis, edema, and total histopathological severity (**Figure 3**). Consistent with previous observations (34), two intraperitoneal administrations of caerulein induced edematous, non-hemorrhagic pancreatitis with no significant changes in hemorrhage and fat necrosis (**Figure 3J**). Under the current protocol, utilizing two doses of caerulein and with tissue collection 5 hours later, no significant hemorrhage and fat necrosis were observed, and inflammation and perivascular infiltration were minimal (**Figures 3J, K**). In contrast, significant increases in acinar necrosis and edema were noted. Stimulation of DMN cholinergic neurons significantly reduced pancreatic edema (**Figure 3M**), and improved the total histological severity, the summation of all other histological scores (**Figure 3N**). In contrast, no changes in edema (**Supplemental Figure 2E**) or total histological severity (**Supplemental Figure 2F**) were observed after blue light (473nm) stimulation in littermate control ROSA-ChR2 mice. Thus, selective activation of brainstem DMN cholinergic neurons regulates disease severity in acute pancreatitis, as evidenced by reductions in serum amylase and pancreatic cytokines, and improved histopathological scoring.

### Selective activation of DMN cholinergic neurons improves pancreatitis *via* a vagus nerve and nicotinic acetylcholine receptor-dependent mechanism

**Figure 4:**
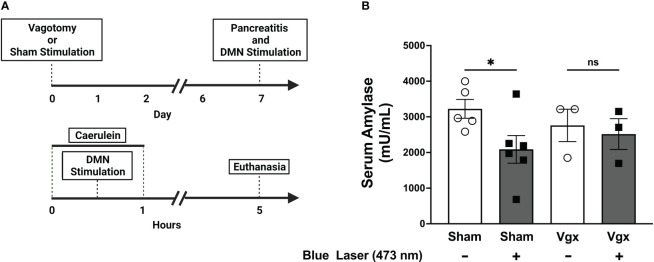
Selective activation of DMN cholinergic neurons fails to reduce serum amylase levels in mice subjected to subdiaphragmatic vagotomy. **(A)** A bilateral subdiaphragmatic vagotomy and transgastric pyloric dilation or sham surgery, consisting of celiotomy and transgastric pyloric dilation only, were performed on ChAT-ChR2-YFP mice. After one week of recovery, pancreatitis was induced with two intraperitoneal injections of caerulein (50 µg/kg). The mice were subjected to optogenetic stimulation with blue light or sham stimulation. The mice were euthanized 4 hours after the final dose of caerulein. **(B)** Optogenetic stimulation with blue light significantly decreases serum amylase levels in sham-operated mice (Sham), but not in mice subjected to subdiaphragmatic vagotomy (Vgx). Data are represented as individual mouse data points with mean ± SEM. One-way ANOVA with Kruskal-Wallis test, (*P ≤ 0.05, n = 3-6), ns, not significant.

Efferent vagus neurons with cell bodies in the DMN provide axonal projections along the vagus nerve and terminate within the pancreas (35). The pancreas also receives projections from splanchnic nerves, and from the enteric nervous system (36, 37). To confirm that the protective effects of DMN cholinergic neurons are propagated *via* the vagus nerve, a subdiaphragmatic vagotomy was performed. Animals were were subjected to bilateral subdiaphragmatic vagotomy and allowed to recover for 7 days. Stimulation of the DMN cholinergic neurons using blue light (473 nm) significantly reduced the serum amylase levels in animals subjected to sham surgery (**Figure 4**). However, in the vagotomized mice lacking a vagus nerve connection between the brainstem and pancreas, DMN stimulation did not decrease serum amylase levels (**Figure 4**). These results provide direct evidence that the protective effects of DMN cholinergic neurons in acute pancreatitis are mediated *via* efferent vagus nerve fibers.

**Figure 5:**
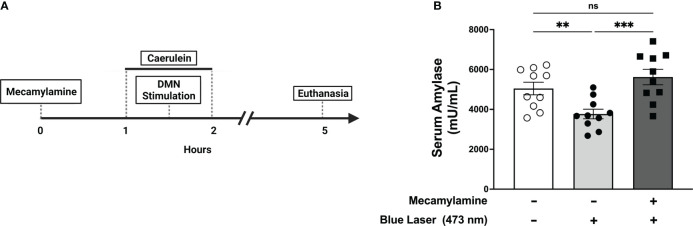
Selective activation of DMN cholinergic neurons reduces serum amylase levels during pancreatitis through a nAChR-dependent mechanism. **(A)** One hour prior to induction of acute pancreatitis, ChAT-ChR2-YFP mice underwent intraperitoneal injection of vehicle or mecamylamine (1 mg/kg) was injected one hour prior followed by the induction of pancreatitis and optogenetic stimulation. The mice were euthanized 4 hours after the final dose of caerulein. **(B)** Optogenetic stimulation with blue light (473 nm, 20 Hz, 25% duty cycle, 8-12 mW, 5 minutes) of the DMN significantly decreases the level of serum amylase compared to no stimulation in the vehicle-treated mice but not in animals receiving mecamylamine. Data are represented as individual mouse data points with mean ± SEM. One-way ANOVA, (**P ≤ 0.01, ***P ≤ 0.001, n = 10, ns, not significant.).

Efferent vagus nerve fibers signal using acetylcholine as a neurotransmitter. We have previously demonstrated that anti-inflammatory effects mediated by efferent vagus nerve signaling require the α7 nicotinic acetylcholine receptor (α7nAChR) (18). To determine the importance of nicotinic acetylcholine receptor signaling in DMN cholinergic neuron-mediated protective effects, we utilized mecamylamine, a non-specific nicotinic acetylcholine receptor antagonist (38–40), to block nicotinic acetylcholine receptor-signaling. Following mecamylamine (1mg/kg, i.p.), stimulation of DMN cholinergic neurons with blue light (473 nm) failed to significantly alter serum amylase levels (**Figure 5**). Together, these results indicate that attenuation of pancreatitis by DMN cholinergic neurons requires vagus nerve signaling to the pancreas *via* the nicotinic acetylcholine receptor.

## Discussion

These studies demonstrate that after pancreatic inflammation is initiated by caerulein, brief and selective activation of cholinergic neurons in the brainstem DMN generate efferent vagus nerve signals which are sufficient to reduce pancreatic injury and the production of pro-inflammatory mediators within the pancreas in a nicotinic acetylcholine receptor-dependent fashion. The selective experimental approach utilized reveals the specific effects of efferent vagus nerve activation on pancreatic inflammation and on the resulting development of acute pancreatitis. The two-injection caerulein model was sufficient to induce the pancreatic tissue injury characteristic of acute pancreatitis, as was demonstrated by the elevation in serum amylase, development of pro-inflammatory cytokines in the pancreas, and histological scoring data. However, the severity of acute pancreatitis generated by two injections of caerulein was restricted primarily to the pancreas and induced a low level of systemic inflammation, as demonstrated by low levels of circulating cytokines. These results indicate that the protective effects mediated by the stimulation of DMN cholinergic neurons result from the direct action of efferent vagus fibers on the pancreas itself and not other organs, such as the spleen.

Selecting a short time point for evaluation following pancreatitis induction was also necessary to isolate the pancreas-specific effects of DMN stimulation. Prior work shows that acinar cells upregulate ICAM-1 expression within 1 hr of exposure to caerulein (41), thereby initiating neutrophil infiltration by 6 hours after injury (the earliest time point evaluated). In experimental pancreatitis models, macrophages infiltrate tissue even later, arriving in the pancreas 24 hours after injury (42). Low levels of infiltrating, non-resident immune cells in the current study were demonstrated by the absence of perivascular infiltration noted in the histology. Therefore, given minimal systemic inflammation and the lack of infiltration noted in experimental tissues, the protective effects of DMN stimulation likely do not involve extra-pancreatic vagus nerve projections, neutrophils, or extra-pancreatic macrophages. These observations are best explained by a direct vagus-nerve-mediated effect on resident pancreatic cells.

Efferent vagus nerve innervation of the pancreas is extensive with the DMN supplying the intrapancreatic ganglia which secondarily innervates other pancreatic cell types and utilizes acetylcholine as a neurotransmitter (43). The observed efficacy of DMN stimulation could arise from acetylcholine-mediated effects on the acinar cell itself, or effects arising from acetylcholine signaling in tissue-resident cells, e.g., macrophages, which widely express nAChR (44). Previous work has shown that increasing ambient ACh by using the cholinesterase inhibitors physostigmine or neostigmine ameliorates the severity of pancreatitis (45). Although these studies would seem to preclude an explanation of the cholinergic anti-inflammatory pathway as originally described (which requires the spleen), the α7nACh receptor is clearly involved as prior work has shown that pretreatment activation of α7nAChR using the specific agonist GTS-21 markedly decreased the development of acute pancreatitis (26).

Recent work describes an increasing variety of parenchymal cell types, in diverse tissues, which express the α7nAChR and, upon activation, confer tissue protection when subjected to stress (46, 47). Although earlier immunohistological work concluded that the acinar cell did not express α7nAChR (48), recent studies have definitively shown acinar cell responses using specific α7nAChR agonists in *in vitro* and *in vivo* models. For example, in experimental pancreatitis, activation of acinar α7nAChR using the specific agonist PNU-282987 restores transcription factor ER, the master regulator of lysosomal synthesis and autophagy, thereby restoring normal lysosomal degradation and preventing/reversing disease at the disease-initiating location (4). It is therefore plausible that in the current study the protective effect occurs primarily at the level of the acinar cell itself and secondarily on immunocompetent cells. Additionally, tissue-resident α7nAChR-expressing macrophages could be a target. However, the resident macrophage population within the pancreatic parenchyma has been poorly characterized, particularly concerning the expression of nicotinic ACh receptor isoforms. Based on the results of the current study, the effector cell type within the pancreas remains unidentified and further study will be necessary to identify the cellular target of DMN stimulation.

In addition to the efferent cholinergic anti-inflammatory pathway, prior work has shown that stimulation of afferent vagus fibers also activates anti-inflammatory activities. Liu and coworkers have shown that the vagus sensory neurons which innervate the deep hindlimb fascia (at the ST-36 site) activate the vagal-adrenal axis *via* the DMN (49). Electroacupuncture *via* the Zuslani acupoint (ST-36) has been shown to ameliorate acute pancreatitis *via* a vagus nerve cholinergic pathway which depends upon the a7nAChR (50). Afferent anti-inflammatory activity of the vagus is also mediated through the splanchnic nerves *via* the celiac and superior mesenteric ganglia (20). Therefore, both afferent and efferent vagus nerve pathways provide anti-inflammatory effects *via* distinct pathways. Whether the effects are synergistic in the intact vagus nerve remains to be determined, although some data have been reported that show stimulation of the intact vagus appears to be less effective than stimulating the cephalic (afferent) trunk or the caudal (efferent) trunk of the transected vagus (20). Thus, further study to determine whether there are differences between afferent and efferent stimulation and which approach can provide the optimum protection in the setting of developing pancreatitis is required.

Although the potential benefits of selective efferent vagus nerve stimulation to control inflammation appear promising, translation into a treatment for human disease constitutes a challenge. Direct stimulation of the DMN is invasive and currently is not amenable for development into clinical practice. However, there has been much recent interest in shaping stimulation waveforms and electrode geometry to directionally activate the vagus nerve (51) and recent work has shown that anodal block technology could be feasible (31). Therefore, the development of this or related technologies for transcutaneous use may allow for the selective activation of vagus efferent fibers in the future. Furthermore, given the pre-clinical data that carotid sheath stimulation containing the vagus nerve is efficacious in acute pancreatitis (27), it is also plausible that more traditional non-selective vagus nerve stimulation could be translated to the treatment of human disease.

Prior study (26) has shown that cervical vagotomy or use of the nAChR receptor antagonist mecamylamine exacerbates caerulein-induced pancreatitis, effects which were not observed in the current study (**Figures 4**, **5**). These differences may be explained by the narrow time window utilized (5 hrs versus 12 hrs) and the limited caerulein exposure (2 doses versus 12 doses), which resulted in less severe pancreatic injury and amylase release. Additionally, the use of subdiaphragmatic vagotomy in the current study compared to cervical vagotomy in the prior study could also contribute to the divergent results noted. In view of these limitations, further study will be required to determine what effects efferent vagal stimulation provides on later stages of pancreatitis, including following a more extensive caerulein dosing protocol. However, given that the evolution of the severe, later-occurring complications of acute pancreatitis (e.g., pancreatic necrosis and pulmonary injury) depends ultimately on the initial acinar injury, it stands to reason that disruption of early disease should mitigate later pathology. It is also expected that curtailing the establishment of pancreatic inflammation by reducing the intra-pancreatic production of IL-6, CXCL1, and MCP-1, would lead to significantly reduced T-cell proliferation and chemotaxis (52), recruitment of neutrophils (53), and macrophages, respectively.

In summary, brief and selective stimulation of vagus nerve efferent fibers during the development of acute pancreatitis activates anti-inflammatory actions within the pancreas, mitigating the severity of tissue damage and reducing the production of pro-inflammatory cytokines and chemokines. These protective effects propagate through the vagus nerve and require nicotinic acetylcholine receptor activity. Although the detailed mechanism of these effects is yet to be delineated, the observed effects of vagus nerve stimulation in acute pancreatitis have significant translational potential for the treatment of a common, highly-morbid disease that currently lacks specific therapy.

## Methods

### Animals

All procedures and experiments were in accordance with NIH guidelines and approved by the Institutional Animal Care and Use Committee and the Institutional Biosafety Committee of the Feinstein Institutes for Medical Research, Northwell Health, Manhasset, NY. The animals were maintained at 25 °C on a 12-hour light-dark cycle. Balanced cohorts of male and female mice, aged 8-12 weeks, were utilized for all experiments. The mice that underwent vagotomy were fasted for 3 hours prior to surgery. At all other times, the mice were allowed free access to food and water. ChAT-cre (B6;129S6-Chattm2(cre)Lowl/J, strain #: 006410) and ROSA-ChR2-YFP (B6.Cg-Gt (ROSA) 26Sortm32 (CAG-COP4*H134R/EYFP)Hze/J, strain #: 024109) were purchased from Jackson Lab and crossed; ChAT-ChR2-YFP were used for experiments along with ROSA-ChR2-YFP in place of littermate controls. Acute pancreatitis was induced by intraperitoneal injections of caerulein (Sigma, C9026) 50 µg/kg of body weight (54). Mecamylamine hydrochloride (Sigma-Aldrich, M9020) 1 mg/kg was administered by intraperitoneal injection 1 hour prior to the induction of acute pancreatitis (26).

### Stimulation of DMN cholinergic neurons

Mice were anesthetized using a mixture of ketamine (144 mg/kg) and xylazine (13 mg/kg) then fixed in a stereotaxic frame with ear bars (David Kopf Instruments) and the left DMN exposed as previously described (28). Briefly, the neck was flexed at approximately 45 degrees and a 1 cm incision was made longitudinally along the posterior midline just inferior to the occipital portion of the skull. The neck muscles were bluntly dissected and reflected laterally, exposing the dura between the base of the skull and the first cervical vertebrae. The dura was then incised using a 23G needle and cerebrospinal fluid (CSF) was absorbed with a Kimwipe (Kimtech Science). With the opening of the fourth ventricle exposed, the obex was used as the cardinal point for locating the DMN. Using stereotaxic guidance, a 200 µm fiber optic cannula (Thorlabs) was lowered to the obex and inserted into the left DMN (0.25 mm lateral, 0.48 mm deep). The sham mice had the fiber optic cannula introduced but were not exposed to laser light. All mice received 5 minutes of pulsed laser light stimulation (20 Hz, 25% duty cycle, 8-12 mW/mm^2^ measured at fiber tip) produced by a function generator (Agilent) and a laser source. Two separate laser sources were utilized: blue (473 nm wavelength, Opto Engine LLC) and yellow (593.5 nm, Opto Engine LLC).

### Heart rate monitoring

During DMN optogenetic stimulation experiments, heart rate was monitored for the duration of stimulation. Data were acquired using the OmniPlex Data Acquisition System (Plexon Inc.) and analyzed *post-hoc* using Spike2 analysis software (Cambridge Electronic Design Ltd). Heart rate was recorded and expressed as beats per minute (BPM).

### Subdiaphragmatic vagotomy and pyloric dilation

The mice were anesthetized using isoflurane (2% induction, 1.5% maintenance). A midline celiotomy was performed and small bowel was placed in the right lower quadrant. A small 4-5 mm incision was made on the greater curvature of the stomach in an area without obvious perforating blood vessels. Vascular dilators were coated in water-based lubricant (Surgilube, HR Pharmaceuticals), then introduced through the gastrotomy and passed across the pyloric sphincter. This was repeated 6 times with successively larger dilators, 1.5-4 mm, increasing 0.5 mm with each dilation. Gastrotomy was closed in a running fashion with an absorbable suture (Vicryl 6.0). The stomach was then retracted inferiorly exposing the esophagus. The anterior and posterior vagus nerves were isolated and ligated just below the diaphragmatic hiatus. Celiotomy was closed in layers with absorbable sutures (Vicryl 5.0) and surgical staples. The sham surgery mice underwent an identical pyloric dilation; however, the vagus nerves were not manipulated. All mice received 1 mL of 37°C, sterile saline subcutaneously prior to being placed in a clean recovery cage and recovered for at least 7 days prior to additional experimentation.

### Tissue processing

Following euthanasia, blood was collected *via* cardiac puncture and serum was isolated *via* centrifugation. For ELISA, the pancreas was immediately placed in tissue protein extraction reagent (Thermo Scientific, 78510) with a protease inhibitor (Thermo Scientific, A32953) and homogenized. The serum and homogenized tissue were stored at -80°C. Pancreatic tissue for histological examination was fixed in neutral buffered formalin (Sigma, HT5011). Paraffin-embedded sections of the pancreas were stained with hematoxylin and eosin (H&E). The samples were evaluated for edema, acinar necrosis, hemorrhage, fat necrosis, inflammation, and perivascular infiltration. A sum of these individual scores was reported as a total score. The severity of acute pancreatitis was graded according to previously established criteria by a pathologist blinded to groups (34). All assessments and scoring were performed by a pathologist blinded to the identity of experiments and experimental groups.

### Assays

Serum amylase activity was measure *via* colorimetric enzymatic assay (Abcam, ab102523). Serum and pancreatic cytokine levels were measured with enzyme-linked immunosorbent assay (ELISA) (Meso Scale Diagnostics, V-Plex).

### Statistical analysis

Statistical analysis was performed using GraphPad Prism (GraphPad Software, v 9.3.0). Normality was determined with Shapiro-Wilk testing. For parametric data sets, two-tailed Student’s T-tests or one-way ANOVA testing was used. For non-parametric data sets, two-tailed Mann-Whitney U-test or Kruskal-Wallis testing was used. Statistical significance was defined as p ≤ 0.05.

## Data availability statement

The raw data supporting the conclusions of this article will be made available by the authors, without undue reservation.

## Ethics statement

The animal study was reviewed and approved by The Feinstein Institutes for Medical Research Institutional Animal Care and Use Committee (IACUC).

## Author contributions

DAT, KT and SC conceived the project, designed experiments, and analyzed data. DAT, TT, AR and SN performed the experiments and subsequent data analysis. VP, LM and DT provided additional comments and contributed to finalizing the manuscript. DAT, MB, KT and SC wrote the manuscript. All authors contributed to the article and approved the submitted version.

## Supplementary material

Supplementary Figure 1 Selective activation of DMN cholinergic neurons in ROSA-ChR2-YFP mice does not invoke efferent vagus nerve activity. **(A)** ROSA-ChR2-YFP mice that do not express a photosensitive channelrhodopsin in ChAT-positive cells, including cholinergic neurons. **(B–D)** Optogenetic stimulation of DMN cholinergic neurons using **(B)** blue light (473nm, 20Hz, 25% duty cycle, 8-12 mW, 5 minutes), **(C)** no light or **(D)** yellow light (593.5nm, 20Hz, 25% duty cycle, 8-12 mW, 5 minutes) does not change heart rate in ROSA-ChR2-YFP mice. **(E)** No change in heart rate is observed after blue or yellow light stimulation. Data are represented as individual mouse data points, which represent an average heart rate over the stimulation period, with mean ± SEM. One way, ****P ≤ 0.0001. ns: not significant.

Supplementary Figure 2 Selective activation of DMN cholinergic neurons in ROSA-ChR2-YFP mice does not reduce the histological severity of acute pancreatitis **(A)** Optogenetic stimulation using blue light or yellow light does not reduce serum amylase levels in ROSA-ChR2-YFP mice. Data are represented as individual mouse data points with mean ± SEM. Mann-Whitney U-test, (P ≥ 0.05 n = 5). **(B, C)** Pancreatic tissue from **(B)** blue and **(C)** yellow light-stimulated mice was collected and stained with H&E 4x. Scale bars **(B, C)** = 200 μm. Compared to non-caerulein-injected mice, mice in both the blue (473 nm, 20 Hz, 25% duty cycle, 8-12 mW, 5 minutes) and yellow light (593.5 nm, 20 Hz, 25% duty cycle, 8-12 mW, 5 minutes) stimulated groups demonstrated the histological characteristic findings of acute pancreatitis, including **(D)** edema and **(E)** total histological severity. Blue or yellow light stimulation does not decrease **(D)** pancreatic edema or **(E)** the total histological severity of acute pancreatitis. Data are represented as individual mouse data points with mean ± SEM. One-way ANOVA with Kruskal-Wallis test. ns: not significant.

Supplementary Figure 3 Optogenetic stimulation of the left dorsal motor nucleus of the vagus (DMN) cholinergic neurons does not alter serum cytokine levels in acute pancreatitis **(A–C)** Low levels of circulating cytokines were observed in mice subjected to caerulein-induced pancreatitis, with a significant increase in **(A)** IL-1β and **(B)** IL-6. Blue (473 nm, 20 Hz, 25% duty cycle, 8-12 mW, 5 minutes) or yellow light (593.5 nm, 20 Hz, 25% duty cycle, 8-12 mW, 5 minutes) stimulation induces a significant decrease in serum **(C)** CXCL-1 but not in **(A)** IL-1β and **(B)** IL-6. Data are represented as individual mouse data points with mean ± SEM. One-way ANOVA, (*P ≤ 0.05, **P ≤ 0.01, ***P ≤ 0.001, ****P ≤ 0.0001, n = 9-12). ns: not significant.
