# Supplementary figures and images for: Optogenetic stimulation of the brainstem dorsal motor nucleus ameliorates acute pancreatitis
Source: Front Immunol. 2023 Apr 25;14:1166212. doi: 10.3389/fimmu.2023.1166212 (PMC10167283; doi:10.3389/fimmu.2023.1166212)

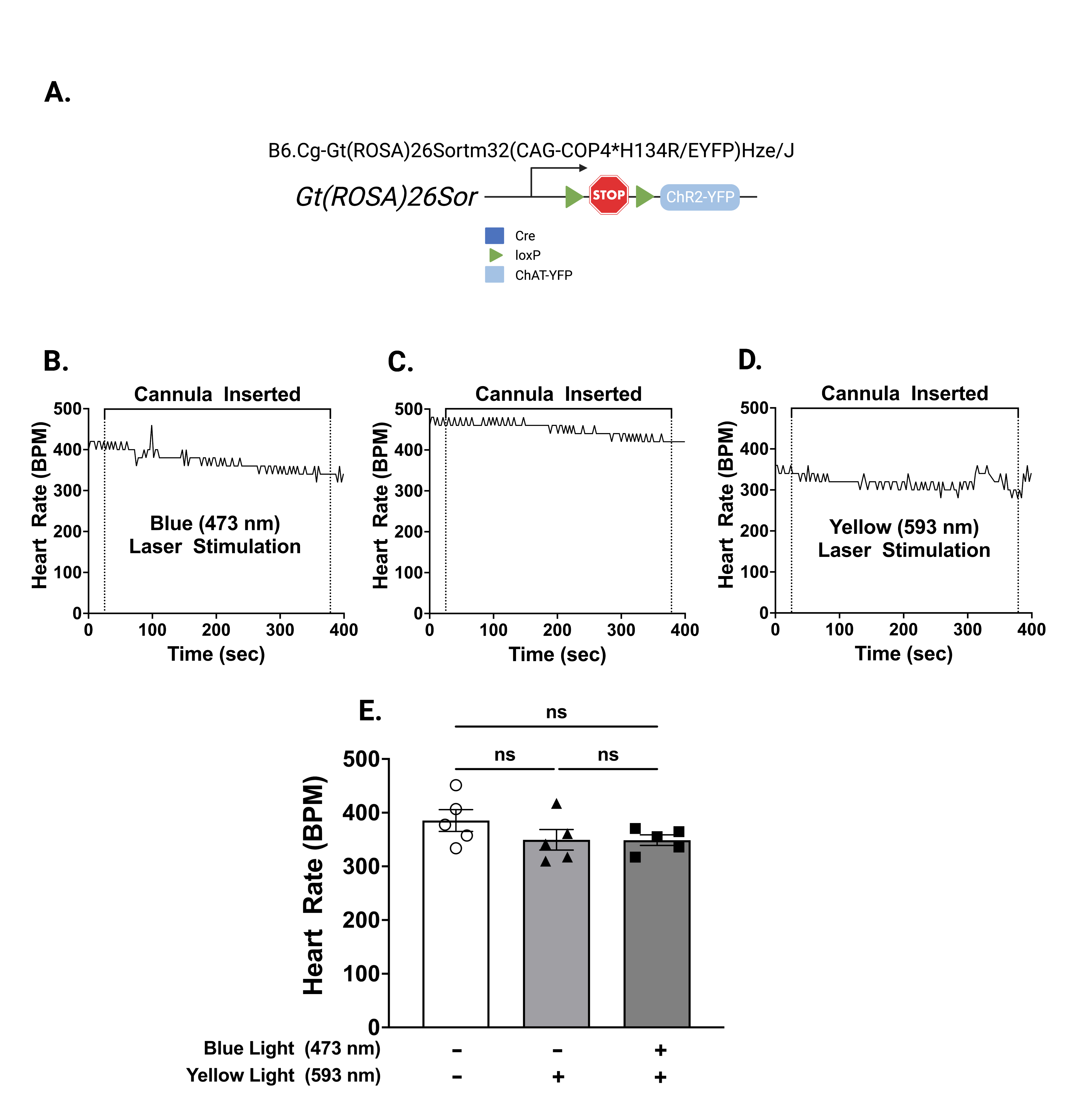

Supplement: Supplementary Figure 1 — Selective activation of DMN cholinergic neurons in ROSA-ChR2-YFP mice does not invoke efferent vagus nerve activity. (A) ROSA-ChR2-YFP mice that do not express a photosensitive channelrhodopsin in ChAT-positive cells, including cholinergic neurons. (B–D) Optogenetic stimulation of DMN cholinergic neurons using (B) blue light (473nm, 20Hz, 25% duty cycle, 8-12 mW, 5 minutes), (C) no light or (D) yellow light (593.5nm, 20Hz, 25% duty cycle, 8-12 mW, 5 minutes) does not change heart rate in ROSA-ChR2-YFP mice. (E) No change in heart rate is observed after blue or yellow light stimulation. Data are represented as individual mouse data points, which represent an average heart rate over the stimulation period, with mean ± SEM. One way, ****P ≤ 0.0001. ns: not significant. [file Image_1.jpg]

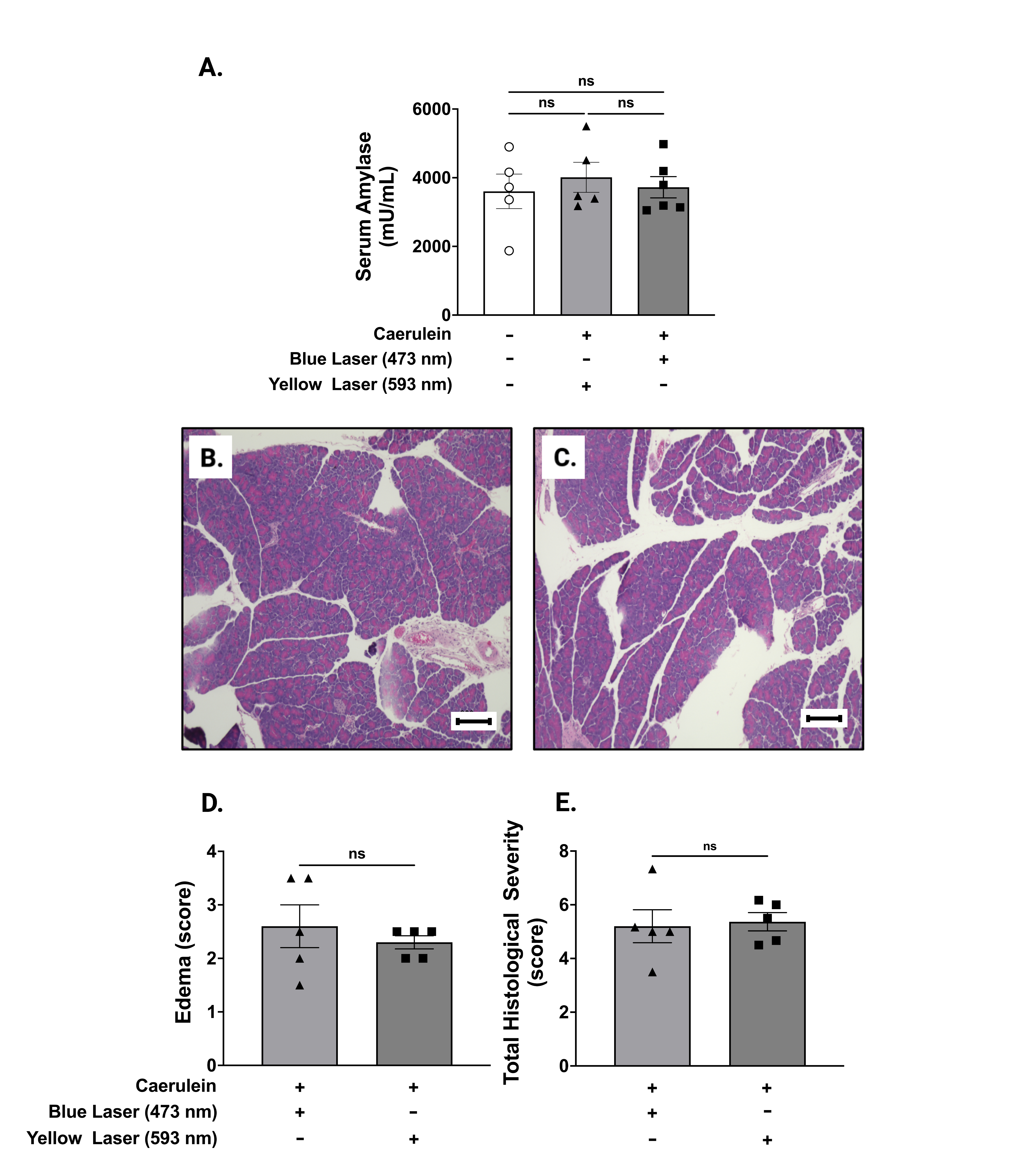

Supplement: Supplementary Figure 2 — Selective activation of DMN cholinergic neurons in ROSA-ChR2-YFP mice does not reduce the histological severity of acute pancreatitis (A) Optogenetic stimulation using blue light or yellow light does not reduce serum amylase levels in ROSA-ChR2-YFP mice. Data are represented as individual mouse data points with mean ± SEM. Mann-Whitney U-test, (P ≥ 0.05 n = 5). (B, C) Pancreatic tissue from (B) blue and (C) yellow light-stimulated mice was collected and stained with H&E 4x. Scale bars (B, C) = 200 μm. Compared to non-caerulein-injected mice, mice in both the blue (473 nm, 20 Hz, 25% duty cycle, 8-12 mW, 5 minutes) and yellow light (593.5 nm, 20 Hz, 25% duty cycle, 8-12 mW, 5 minutes) stimulated groups demonstrated the histological characteristic findings of acute pancreatitis, including (D) edema and (E) total histological severity. Blue or yellow light stimulation does not decrease (D) pancreatic edema or (E) the total histological severity of acute pancreatitis. Data are represented as individual mouse data points with mean ± SEM. One-way ANOVA with Kruskal-Wallis test. ns: not significant. [file Image_2.jpg]

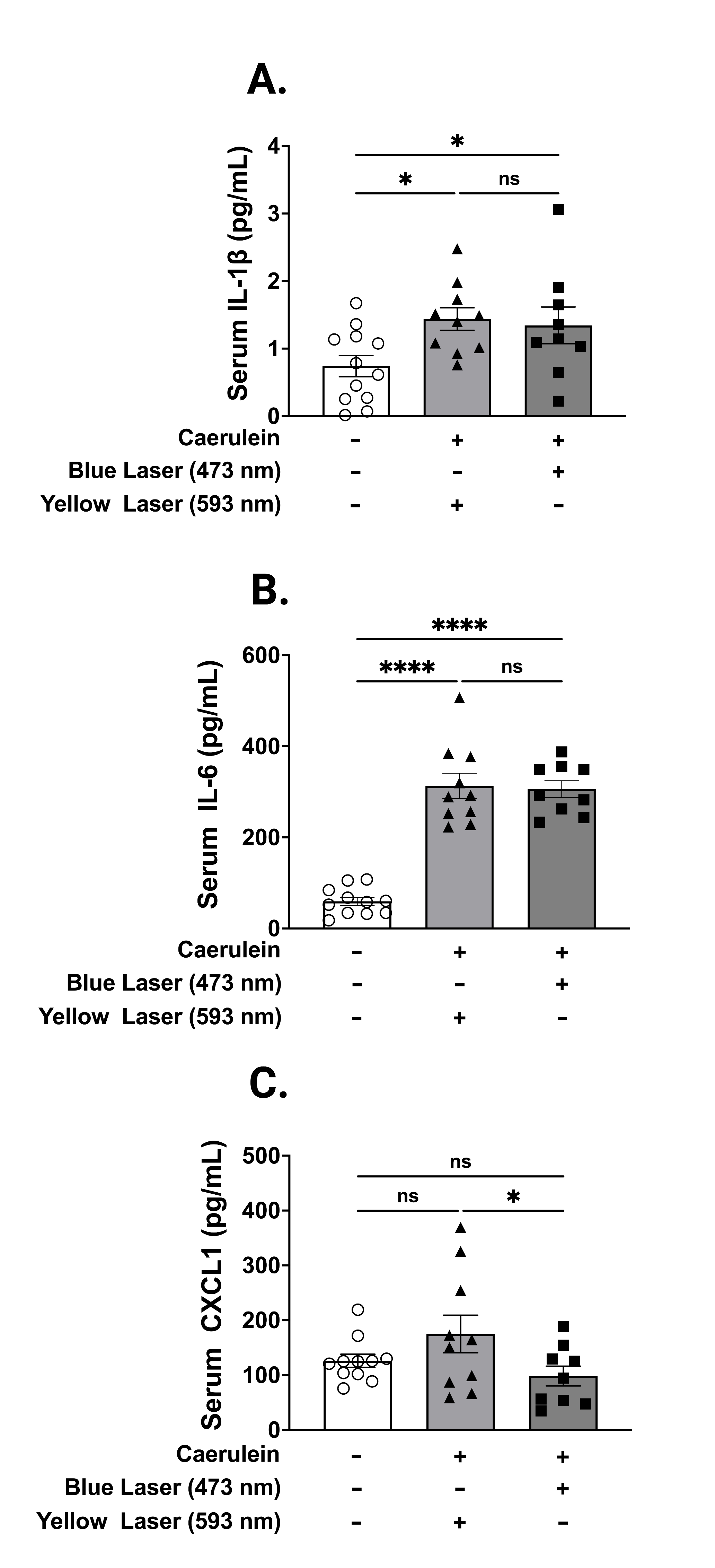

Supplement: Supplementary Figure 3 — Optogenetic stimulation of the left dorsal motor nucleus of the vagus (DMN) cholinergic neurons does not alter serum cytokine levels in acute pancreatitis (A–C) Low levels of circulating cytokines were observed in mice subjected to caerulein-induced pancreatitis, with a significant increase in (A) IL-1β and (B) IL-6. Blue (473 nm, 20 Hz, 25% duty cycle, 8-12 mW, 5 minutes) or yellow light (593.5 nm, 20 Hz, 25% duty cycle, 8-12 mW, 5 minutes) stimulation induces a significant decrease in serum (C) CXCL-1 but not in (A) IL-1β and (B) IL-6. Data are represented as individual mouse data points with mean ± SEM. One-way ANOVA, (*P ≤ 0.05, **P ≤ 0.01, ***P ≤ 0.001, ****P ≤ 0.0001, n = 9-12). ns: not significant. [file Image_3.jpg]
